# Direct letters to relatives at risk of hereditary cancer—a randomised trial on healthcare-assisted versus family-mediated risk disclosure

**DOI:** 10.1038/s41431-025-01922-w

**Published:** 2025-07-31

**Authors:** Hans Ehrencrona, Anna Öfverholm, Carolina Hawranek, Lovisa Lovmar, Sara Svensson, Sigrid Wennstedt, Barbro Hellquist, Anna Rosén

**Affiliations:** 1 Division of Clinical Genetics, Department of Laboratory Medicine, Lund University, Lund, Sweden; 2 Department of Clinical Genetics, Pathology and Molecular Diagnostics, Skåne University Hospital, Lund, Sweden; 3 Department of Oncology, Institute of Clinical Sciences, Sahlgrenska Academy, University of Gothenburg, Göteborg, Sweden; 4 Department of Diagnostics and Intervention, Oncology, Umeå University, Umeå, Sweden; 5 Department of Clinical Genetics and Genomics, Sahlgrenska University Hospital, Gothenburg, Sweden; 6 Department of Medical Biosciences, Umeå University, Umeå, Sweden; 7 Department of Clinical Microbiology, Umeå University, Umeå, Sweden

**Keywords:** Genetics research, Cancer genomics, Genetic counselling, Cancer prevention, Randomized controlled trials

## Abstract

Observational studies suggest that direct contact from healthcare to at-risk relatives may increase genetic counselling (GC) uptake as compared to family-mediated risk disclosure, but randomised controlled trials (RCTs) are lacking. This study assessed whether the offer of direct letters to relatives at risk of hereditary breast and ovarian cancer (HBOC) or Lynch syndrome increases GC uptake compared to family-mediated communication alone. Between 2020 and 2023, probands were randomly assigned to family-mediated disclosure (control) or family-mediated disclosure plus the offer of sending direct letters to at-risk relatives (intervention). The primary outcome was GC uptake within 12 months, measured as the proportion of eligible relatives at risk contacting a Swedish cancer genetics clinic. In total, 165 families (median: 4 eligible relatives, range: 1–26) were randomised to control (*n* = 79) or intervention (*n* = 86). GC uptake was 67% in controls and 71% in the intervention group (*P* = 0.23). After adjusting for predefined variables and covariates, there was still no significant difference between groups (OR: 1.24, CI: 0.79–1.95, *P* = 0.34). Distant relatives had lower uptake than first-degree relatives (OR: 0.27, CI: 0.18–0.40, *P* < 0.001), while female relatives had higher uptake than males (OR: 2.17, CI: 1.50–3.12, *P* < 0.001). This is the largest RCT so far investigating direct letters to relatives. GC uptake was high in both groups, and the intervention of direct letters did not show superiority over family-mediated communication alone. Direct letters to relatives may complement family-mediated disclosure in certain situations, but should not be implemented as a general procedure in cancer genetics practices.

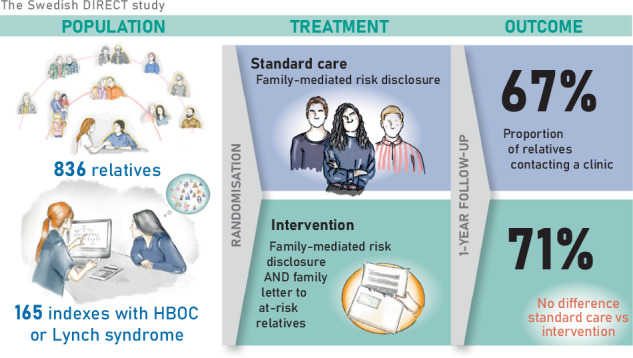

## Introduction

Advancements in genetic testing have facilitated the identification of germline pathogenic variants (PVs) in cancer predisposition genes, with implications for both patients and their at-risk relatives (ARRs). This is particularly significant for PVs in *BRCA1, BRCA2* and *PALB2*, causing hereditary breast and ovarian cancer syndrome (HBOC), and mismatch repair genes causing Lynch syndrome. Surveillance and risk-reducing surgeries are associated with >90% reduction in cancer incidence and significantly decreased cancer mortality for female *BRCA1/BRCA2* carriers [1]. Similarly, colonoscopy surveillance and risk-reducing hysterectomy and salpingo-oophorectomy mitigate cancer morbidity and mortality in Lynch syndrome (*MLH1, MSH2, MSH6, PMS2*) [2, 3].

In most European countries, no specific guidelines on risk disclosure to ARRs exist [4]. Current clinical practice relies on proband-mediated information disclosure to ARRs, where studies show ~35% uptake of genetic counselling (GC) among ARRs, but with a large difference in uptake between different studies and different subgroups [5–7]. While 94% of probands disclose to at least one ARR and 70% of ARRs receive information about the familial PV [8], probands experience various barriers while also recognising their responsibility to share information [9–12]. Barriers include fear of causing distress, privacy concerns, relationship distance and contact information availability [13]. Gender [5] and degree of kinship [14] influence both disclosure behaviour and cascade testing uptake, with higher rates of disclosure to female and first-degree relatives [8]. Information accuracy may be compromised when transmitted solely through probands rather than healthcare professionals (HCPs) [15].

A complementary strategy to family-mediated risk disclosure is direct contact with the ARRs by the genetic HCP, either by phone, letter, or email. Sending direct letters has been implemented as a general procedure in some countries. In Denmark, unsolicited letters have been used for decades as a method of risk disclosure to relatives at risk of Lynch syndrome [16]. In the Netherlands, the guidelines for hereditary cancer syndromes allow HCP-assisted contact to ARRs by letter (if the proband lacks contact details to ARRs and consents to the procedure) [17]. In France, probands with a serious genetic condition with available preventive measures are legally bound to either inform ARRs themselves or to authorise a HCP to do it for them (with HCP-assisted contact by letter) [18].

The first meta-analysis of healthcare-assisted risk disclosure to ARRs in HBOC and Lynch families reported that direct contact significantly increased uptake of GC, as compared to family-mediated risk disclosure (63% [95% CI: 49–75] vs. 35% [95% CI: 24–48]) [5]. However, studies cited in this meta-analysis, and in other reviews evaluating cascade GC and testing of hereditary cancer [6–8], present important limitations. First, they are mostly observational, non-randomised evaluations and secondly, they are heterogeneous in terms of study context and outcome measures, which hamper cross-study comparisons [19]. To move the field forward, well-powered randomised studies with a control group are needed.

The overall aim of this prospective, multicentre, randomised study was to evaluate whether healthcare-assisted disclosure increases GC uptake in ARRs compared to family-mediated disclosure alone, focusing on high-risk cancer predisposition genes (*BRCA1, BRCA2, PALB2, MLH1, MSH2, MSH6, PMS2)*.

## Subjects and methods

### Study context

The management of genetic testing results and consequent risk information is not explicitly addressed in Swedish national legislation [4]. However, as noted in the preparatory works to the Genetic Integrity Act (2006:351), HCPs may disclose genetic test results to ARRs with proband consent, with consideration of individual circumstances.

Specialised healthcare investigating hereditary cancer predisposition syndromes is offered at cancer genetics clinics at Sweden’s university hospitals. All clinics were invited, and four met criteria for participation (see study protocol [20] for details). One clinic had poor adherence to the study protocol, failed progression criteria and poor quality at audit. This site was closed after a year, and probands from this site were not included in any of the results.

### Trial design and participants

The DIRECT study was a pragmatic, open-label, multicentre, randomised controlled trial, ClinicalTrials.gov identifier NTC04197856, with protocol previously published [20]. Patients over 18 years at cancer genetics clinics were pre-screened and invited to participate if they were eligible for genetic screening or predictive testing for suspected HBOC (the genes *BRCA1, BRCA2, PALB2*) or Lynch syndrome (the genes *MLH1, MSH2, MSH6, PMS2*).

The inclusion criteria were (a) signed informed consent, (b) a PV in a gene associated with HBOC or Lynch syndrome, or a negative genetic screening but belonging to a family fulfilling clinical criteria for familial breast cancer or familial colorectal cancer and (c) having at least one eligible ARR, i.e. a family member with no previous contact with a cancer genetics clinic who was deemed to be recommended GC within a year. Inclusion and randomisation were done after the test results were known, but before post-test counselling. The included participants were both cancer patients who underwent genetic screening and individuals who underwent predictive testing, collectively referred to as ‘probands’ onwards. The probands were randomly assigned in a 1:1 ratio, stratified by study site, gender, age group and family diagnosis. In this article, we report the outcomes in HBOC and Lynch syndrome families. The outcomes of the full cohort are available at our data repository.

### Standard care

All probands received standard care according to current local clinical practice, which included post-test GC with an HCP who also encouraged family-mediated risk disclosure to ARRs. Standard care included written information produced by the different sites, and the content was largely similar across sites and over time. The printed information included details on hereditary cancer, cascade testing, risk management and contact information for cancer genetics clinics.

### Definition and identification of eligible ARRs

At the post-test counselling session, the proband, together with the involved HCPs, identified eligible ARRs >18 years at the end of follow-up, with no previous contact with a Swedish cancer genetics clinic. The relatives who were eligible as ‘at-risk’ were adult male and female relatives who, according to the involved HCP, could be offered cascade GC and testing for the familial PV within 12 months. In general, the living ARRs with the closest (genetic) relationship to the proband were considered eligible and offered cascade GC and testing before subsequent relatives were eligible. However, more distant (non-first-degree) ARRs were considered eligible if the first-degree relative was deceased or, due to other reasons, was unavailable for cascade GC and testing. During our weekly study meetings, the involved HCP consulted the study team if they were unsure about how to define eligible ARRs, but the final decision on who to list as an eligible ARR and thus to be recommended GC within a year was up to the local HCP. Contact details of ARRs in both control and intervention groups were retrieved in collaboration between the proband and the HCP. At times, the HCP and the proband collaboratively employed official publicly available sources to identify ARRs’ contact details.

### Intervention

Probands in the intervention group, as well as the control group, received standard care and both arms did the listing of eligible ARRs as outlined above. In addition, the intervention group was offered the service of the HCP sending a direct letter to their eligible ARRs. The proband approved or denied contact with each eligible ARR. The direct letter included information about the specific familial investigation and the possible implications for them and their family. Templates of direct letters are published in the protocol [20].

The letters were sent ~1 month after the proband had received post-test GC. In some cases, the timing was adjusted according to the proband’s preference. If a listed ARR had already contacted the cancer genetics clinic before the distribution of letters, a letter was not sent to that specific ARR. Distribution of letters was paused before national holidays to reduce the possibility of delayed contact with an available HCP. The letters were sent via registered mail, requiring proof of identity for the recipients to retrieve the letters. The cancer genetics clinic received the letter in return, should the addressee fail to collect it within 2 weeks.

### Outcomes: uptake of genetic counselling in eligible at-risk relatives

In this study, we used ARRs’ contact with a cancer genetics clinic as a proxy for the uptake of GC. The primary outcome was the proportion of eligible ARRs contacting a Swedish cancer genetics clinic within 12 months after the proband received post-test counselling. This follow-up period was chosen because we believe that, from a clinical perspective, the eligible ARRs should preferably have received GC within this period of time. The outcome ‘contact’ was defined as the ARR having an interaction with a Swedish cancer genetics clinic (by phone, video, electronic communication, or a physical meeting) that was documented in the ARR’s patient record or the family pedigree during the follow-up period. Outcome data were collected by the study coordinator at each site, by checking local patient data registries and/or asking the other national collaborating units if they had any registered contact with the ARR within 12 months after inclusion of the proband. Thus, all Swedish cancer genetics clinics were involved in providing data for the primary outcome. Only a few ARRs declined GC after contacting the clinics at the participating study sites (HE, AÖ, AR, personal communication).

The following study-related information on ARRs was reported at the family level: total number of eligible ARRs who had a documented contact with any Swedish cancer genetics clinic within 12 months, and total number of eligible ARRs at the time of inclusion. ARRs’ gender (female and male) and degree of kinship (first-degree and more distant) were detailed. The total number of ARRs, including those who initially lacked contact details, was also reported. Difference in uptake was evaluated both for eligible ARRs (for which contact details were available, main analysis) and for all ARRs (eligible ARRs and ARRs to whom the proband lacked contact details).

### Psychosocial outcomes

Questionnaires were administered to the probands at two time points, baseline at inclusion and ~6 months later. Questionnaires used validated instruments measuring generic health-related quality of life (RAND-36) [21], anxiety (State-Trait Anxiety Inventory, STAI) [22] and cancer worry (CWS) [23]. Non-respondents received one reminder per questionnaire. In this paper, we compared the emotional well-being component of RAND-36, the STAI-S and the 8-item CWS between intervention and control, both at baseline and 6 months follow-up.

### Statistical methods

To detect a 12.5% difference in uptake of GC between groups, we estimated a need to recruit outcome data for 490 ARRs. Main outcome measures were evaluated using chi^2^-tests and logistic regression. As grouping effects at the family level may affect outcomes, we expanded the logistic model to a generalised linear mixed model (GLMM) with a logit link. Multivariable GLMMs were adjusted for the stratification variables (gender and age group of the proband, family diagnosis and study site) as well as degree of kinship and gender of the ARRs. The Shapiro-Wilk test was used to test for non-normality. The Wilcoxon rank sum test was used for comparison of family size and the psychosocial measurements. *P* values < 0.05 were considered significant, and all analyses were conducted using R (version 4.4.0) [24].

## Results

### Study population

**Fig. 1:**
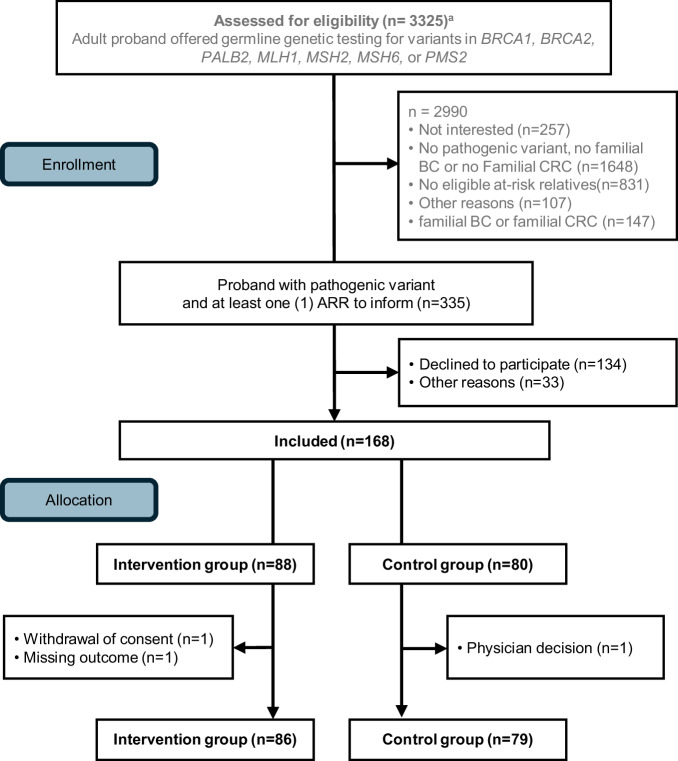
CONSORT diagram. ARR at-risk relative, BC breast cancer, CRC colorectal cancer, HBOC hereditary breast and ovarian cancer syndrome. ^a^Since randomisation was performed before post-test counselling, probands were screened for eligibility before genetic test results were available.

Between January 1st, 2020, and October 31st, 2023, 3325 patients were screened for eligibility at the time of offering genetic testing. When test results were available, 335 had HBOC or Lynch syndrome and at least one uninformed ARR, and 168 of those were included in the study (Fig. 1). Outcome data were available for 836 eligible ARRs of 165 probands. Family size ranged from 1 to 26 eligible ARRs with a non-normal distribution (*P* < 0.001), and there was no difference in median family size between the intervention and control group (*P* = 0.44). Median number of eligible ARRs per family was 4 in both arms. Sociodemographic and clinical characteristics of participants are found in Table 1.

**Table 1:** Characteristics of the allocated probands with HBOC or Lynch syndrome.

	Total (*n* = 165)	Intervention (*n* = 86)	Control (*n* = 79)
**Proband age** Median (min-max)		56 (21–90)	59 (29–83)
**ARR per proband** Median (min-max)		4 (1–26)	4 (1–17)
Proband sex, No. (%)			
Female	128	67 (78%)	61 (77%)
Male	37	19 (22%)	18 (23%)
Proband age, No. (%)			
<50 years	48	34 (40%)	14 (18%)
≥50 years	117	52 (60%)	65 (82%)
Study site, No. (%)			
A	70	41 (48%)	29 (37%)
B	39	15 (17%)	24 (30%)
C	56	30 (35%)	26 (33%)
Referral reason, No. (%)			
Genetic screening	118	65 (76%)	53 (67%)
Predictive testing	47	21 (24%)	26 (33%)
Family diagnosis, No. (%)			
HBOC	124	66 (77%)	58 (73%)
Lynch syndrome	41	20 (23%)	21 (27%)
Genetic finding, No. (%)			
*BRCA1*	67	35 (41%)	32 (41%)
*BRCA2*	41	23 (27%)	18 (23%)
*PALB2*	16	8 (9%)	8 (10%)
*MLH1*	5	2 (2%)	3 (4%)
*MSH2*	10	5 (6%)	5 (6%)
*MSH6*	18	9 (10%)	9 (11%)
*PMS2*	8	4 (5%)	4 (5%)
Educational attainment, No. (%)			
<9 years	8	3 (3%)	5 (6%)
Compulsory (9 years)	22	14 (16%)	8 (10%)
Upper secondary (12 years)	26	17 (20%)	9 (11%)
University ≥1 year	32	12 (14%)	20 (25%)
University degree	53	28 (33%)	25 (32%)
NA	24	12 (14%)	12 (15%)

*ARR* at-risk relative, *HBOC* hereditary breast and ovarian cancer syndrome.

### Crude uptake of genetic counselling

**Fig. 2:**
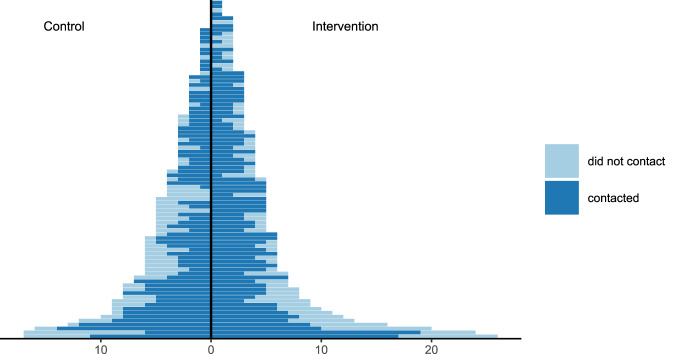
Number of ARRs per family who contacted (dark blue) and did not contact (light blue) a cancer genetics clinic within a year. ARR at-risk relative.

The number of ARRs per family who contacted or did not contact a cancer genetics clinic within a year is depicted in Fig. 2. Crude uptake of GC in ARRs was 67% in the control group and 71% in the intervention group (*P* = 0.23). In pre-planned subgroup analyses based on proband’s gender, age group, study site, referral reason, family diagnosis, ARRs´ gender and degree of kinship, there was no significant difference in crude uptake of GC between study groups (Table 2). Uptake of GC in female ARRs in HBOC families was 148/171 (87%) in the intervention and 114/142 (80%) in the control group, and in Lynch families 46/64 (72%) in the intervention and 44/58 (76%) in the control group. Corresponding uptake of GC in male ARRs in HBOC families was 91/145 (63%) in the intervention and 74/124 (60%) in the control group, and in Lynch families 40/77 (52%) in the intervention and 22/55 (40%) in the control group.

**Table 2:** Crude uptake of genetic counselling (GC) in the intervention and control group.

	Intervention	Control	Chi^2^-test
	*n ARRs contacting a cancer genetics clinic/n eligible ARRs (%)*	*n ARRs contacting a cancer genetics clinic/n eligible ARRs (%)*	*P*
Total	325/457 (71%)	254/379 (67%)	0.23
Proband sex			
Female	242/327 (74%)	208/313 (66%)	0.05
Male	83/130 (64%)	46/66 (70%)	0.51
Proband age			
<50	102/138 (74%)	26/36 (72%)	1
≥50	223/319 (70%)	228/343 (66%)	0.39
Study site			
A	168/235 (71%)	75/104 (72%)	1
B	67/95 (71%)	88/137 (64%)	0.39
C	90/127 (71%)	91/138 (66%)	0.47
Referral reason			
Genetic screening	245/341 (72%)	188/281 (67%)	0.21
Predictive testing	80/116 (69%)	66/98 (67%)	0.33
Family diagnosis			
HBOC	239/316 (76%)	188/266 (71%)	0.21
Lynch syndrome	86/141 (61%)	66/113 (58%)	0.77
ARR sex			
Female	194/235 (83%)	158/200 (79%)	0.41
Male	131/222 (59%)	96/179 (54%)	0.33
Degree of kinship			
First-degree	198/245 (81%)	155/211 (73%)	0.08
≥Second-degree	127/212 (60%)	99/168 (59%)	0.93

*ARR* at-risk relative, *HBOC* hereditary breast and ovarian cancer syndrome.

### Generalised linear mixed model

**Fig. 3:**
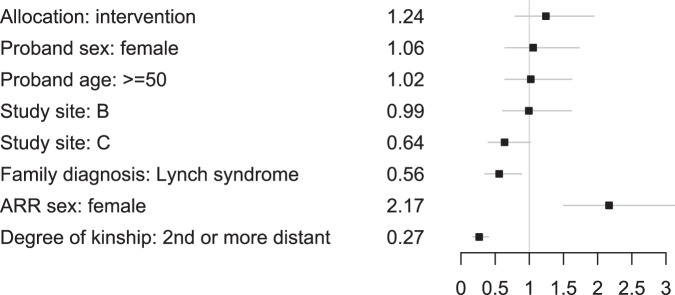
Odds ratios and 95% confidence intervals from the multivariable generalised linear mixed model for the uptake of GC. Reference levels used in the model: Allocation: control group, Proband sex: male, ARR sex: male, Kinship: 1st degree relative, Study site: A, Proband age group: <50, and Family diagnosis: HBOC. ARR at-risk relative, HBOC hereditary breast and ovarian cancer syndrome.

After adjusting for the predefined stratifying variables (proband gender, age group, study site and family diagnosis), and the covariates ARR’s gender and kinship, the randomisation groups still showed no difference in GC uptake (OR: 1.24, CI: 0.79–1.95, *P* = 0.34). Female ARRs had a significantly higher uptake than male ARRs (OR: 2.17, CI: 1.50–3.12, *P* < 0.001). Also, distant relatives had a significantly lower uptake than first-degree relatives (OR: 0.27, CI: 0.18–0.40, *P* < 0.001). ARRs in families with Lynch syndrome had a significantly lower uptake than those in families with HBOC (OR: 0.56, CI: 0.35–0.89, *P* = 0.01) (Fig. 3, Table 3).

**Table 3:** Odds ratios with 95% CI and *p* values of uptake of genetic counselling in ARRs at risk of HBOC and Lynch syndrome, generated from univariable and multivariable generalised linear mixed model (GLMM) of different sociodemographic and clinical predictors.

		Univariable			Multivariable		
		OR	95% CI	*P*	OR	95% CI	*P*
Exposure	Control	1			1		
	Intervention	1.18	0.77–1.81	0.45	1.24	0.79–1.95	0.34
Proband sex	Male	1			1		
	Female	1.31	0.85–2.04	0.22	1.06	0.65–1.73	0.83
Proband age	<50	1			1		
	≥50	0.72	0.45–1.16	0.18	1.02	0.64–1.62	0.93
Study site	A	1			1		
	B	0.80	0.48–1.32	0.38	0.99	0.61–1.62	0.98
	C	0.82	0.52–1.29	0.38	0.64	0.40–1.02	0.06
Family diagnosis	HBOC	1			1		
	Lynch syndrome	0.53	0.36–0.77	<0.001	0.56	0.35–0.89	0.01
ARR sex	Male	1			1		
	Female	3.58	2.55–5.02	<0.001	2.17	1.50–3.12	<0.001
Degree of kinship	First-degree	1			1		
	≥Second-degree	0.19	0.12–0.27	<0.001	0.27	0.18–0.40	<0.001

*ARR* at-risk relative, *HBOC* hereditary breast and ovarian cancer syndrome.

### Analysis including ARRs with unknown contact details

In an additional analysis of the 165 probands with a PV causing either HBOC or Lynch syndrome, we included the 122 ARRs for whom contact details were unknown (and follow-up not possible) to the denominator. The control group had a significantly higher percentage of ARRs for whom contact details were unknown (70/449, 15.6%), as compared to the intervention group (52/509, 10.2%) (*p* = 0.017). Crude GC uptake in this additional analysis was 325/509 (64%) in the intervention group and 254/449 (57%) in the control group. The difference in uptake between the intervention and control group was significant in the crude chi^2^-test (*P* = 0.03) and univariable GLMM (OR: 1.60, CI: 1.01–2.53, *P* = 0.04) but not in the multivariable GLMM (OR 1.37, CI: 0.86–2.18, *P* = 0.18).

### Psychosocial measurements

The response rate was 84% (intervention *n* = 72, control *n *= 67) for the baseline questionnaire and 81% (intervention *n* = 66, control *n* = 68) for the 6-month follow-up questionnaire. At both baseline and follow-up, there was no difference in median score in emotional well-being (RAND-36), anxiety (STAI-S) or CWS between the intervention and control group. (RAND-36 emotional well-being baseline 68 vs 72, *P* = 0.94, follow-up 80 vs 76, *P* = 0.06; STAI-S baseline 39.5 vs 38, *P* = 0.96, follow-up 35 vs 36, *P* = 0.25; CWS baseline 16.5 vs 17, *P* = 0.89, follow-up 16 vs 16.5, *P* = 0.69).

### Distribution of direct letters in the intervention group

**Fig. 4:**
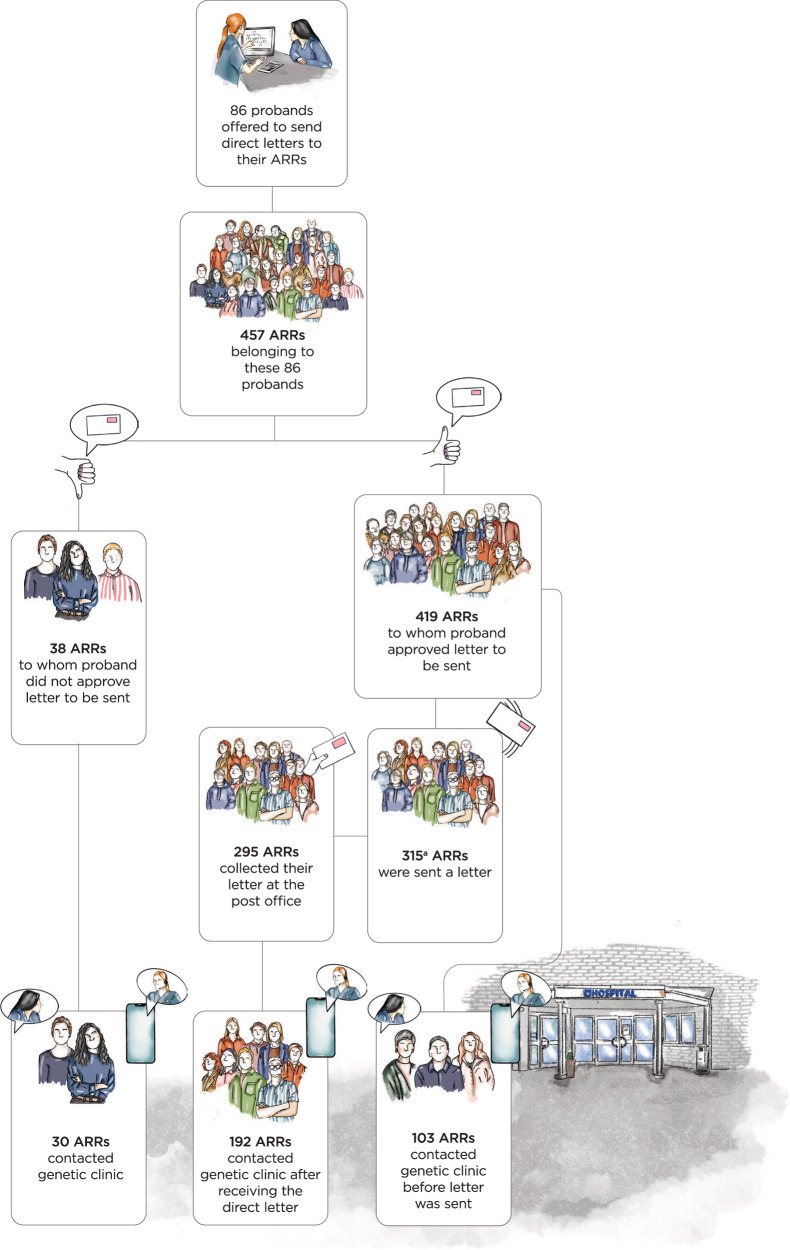
Distribution of direct letters to ARRs in the intervention group. ARR at-risk relative. ^a^One proband first approved letters to be sent to all her eligible ARRs, but later reported that one of her ARRs did not want to receive a letter, and therefore one approved letter was not sent.

Out of 245 eligible first-degree ARRs and 212 distant ARRs, the proband accepted the distribution of letters to 219 (89%) and 200 (94%), respectively (Fig. 4). Among the pre-approved recipients, 84 (39%) first-degree and 19 (10%) distant ARRs contacted a cancer genetics clinic before letters were sent. Of all female pre-approved letter recipients, 68 of 211 (32%) contacted a cancer genetics clinic before the letter was sent, and of the 208 male ARRs, the corresponding number was 35 (17%).

In total, 315 direct letters were sent to 134 first-degree and to 181 distant ARRs, 295 letters (94%) were collected at the post office by the recipients. Of the 295 ARRs who collected their direct letter, 192 (65%) contacted a clinic within the follow-up time. Of all ARRs who collected a letter, there was a significant difference in uptake of GC between first-degree ARRs as compared to distant ARRs (91/126 vs 101/169, *P* = 0.04) and female ARRs as compared to male ARRs (107/131 vs 85/164, *P* < 0.001). Of note, 30 of the 38 ARRs (79%) to whom the proband did not approve contact by letter contacted a cancer genetics clinic within 12 months. The majority of the ARRs (26 out of 38) to whom the proband did not approve contact were first-degree ARRs, and of these 88% (23 out of 26) contacted the cancer genetics clinic within a year. Among the distant ARRs to whom a letter was not approved, the GC uptake was 7 out of 12.

## Discussion

In this randomised controlled trial, we could not demonstrate any significant difference in GC uptake in ARRs between a group offered healthcare-assisted disclosure (direct letters to ARRs) and a control group relying on standard care with family-mediated disclosure (crude uptake 71% vs. 67%, *P* = 0.23; adjusted analysis OR: 1.24, CI: 0.79–1.95, *P* = 0.34).

Observed lower GC uptake among male relatives, distant relatives, and those at risk for Lynch syndrome is consistent with previous research [5, 6, 25]. Notably, the intervention in our study with direct letters did not increase the uptake in these subgroups.

Our findings align with two studies performed in a clinical genetics setting where sending direct letters had no effect on uptake of GC, including a randomised trial on inherited cardiac conditions [26] and a study on *BRCA1/2* families in the Netherlands [17]. While previous systematic reviews and meta-analyses suggest an increased uptake after direct contact initiatives [5, 6], the cited articles primarily rely on heterogeneous observational studies. These studies typically lack control groups; hence, the evaluation is more susceptible to biases, which makes it more difficult to isolate the true impact of the intended intervention from other study-related components. Interventions to improve cascade GC aim to induce a behavioural change in the proband and/or the ARR and/or the HCPs, which requires an in-depth understanding of which components that in fact are the ‘active ingredients’. Ballard et al. pinpoint in their SWiM-analysis of previous GC-interventions that they have often been designed using an implicit common-sense approach consisting of personal experience and a brief analysis of the behaviour. Instead, the authors suggest that the planning of a behavioural change intervention should preferably include a thorough analysis and a model explaining how, when and why an intervention does, or does not, work. Factors such as e.g. detailed listing of ARRs, which can be an intervention in itself [7, 27], may have confounded results in previous studies. We acknowledge that the current study may also have study-related biases, such as behaviour being affected by the listing procedure and/or awareness of being observed as part of a study (the Hawthorne effect). Whereas these factors may contribute to the high uptake in the control group, our randomised design allows for a valid assessment of the added value of offering direct letters, as this was the only component that differed between groups.

The relatively high GC uptake in both our intervention and control groups could also reflect an already effective cascade testing procedure in the Swedish healthcare system, characterised by universal access, subsidised care, and high public trust [28, 29]. This contrasts with settings where cost and insurance coverage are significant barriers [10]. Unlike studies primarily from the US [8], where only 43% of informed ARRs completed testing, our study showed that 65% of direct letter recipients sought counselling (Fig. 4), with few declining subsequent GC and testing (HE, AÖ, AR, personal communication). This suggests that healthcare accessibility may be more crucial than solely focusing on improved methods for disclosure.

In a qualitative study within the trial, 17 probands from both study groups were interviewed [30]. They personally informed all their close relatives, and those in the intervention group also described that they accepted to have direct letters sent by the HCP to all their ARRs. Risk disclosure to distant relatives was less predictable, ranging from direct personal communication to delegating the task to another family member or the HCP by approving the use of direct letters. In another qualitative study within the trial, interviews were conducted with 14 ARRs who had received a direct letter [31]. Many reported that a family member had informed them in advance about the letter, while those who had not been pre-notified described having a more distant relationship with the proband. The ARRs expressed that family communication was beneficial when handling the experience of receiving risk information, and that giving prior notice of the direct letter was considered the appropriate thing to do.

In the current study, the probands in the intervention group accepted direct letters to be sent to the majority (>90%) of their ARRs (Fig. 4). However, 38% of first-degree and 10% of distant ARRs contacted a cancer genetics clinic before any letters were sent to them, suggesting that family-mediated communication plays a central role, particularly for first-degree ARRs. This is also reflected in the fact that the uptake was even higher in the 38 ARRs to whom the proband declined the offer to have a direct letter sent than among ARRs receiving the direct letter. Subgroup analysis of distant ARRs—who had a lower uptake in this study—did not demonstrate increased uptake in the intervention as compared to the control group. No adverse psychological effects of the intervention were observed in the probands, a finding corroborated by a similar study [26]. While direct contact seems psychologically safe [32, 33] and appears well-accepted by relatives [31, 34], the moral duty of information sharing remains ethically complex [9, 35–41].

### Limitations and strengths

Study limitations include potential selection bias, as those opposed to risk disclosure or direct letters may have declined participation. The detailed ARR listing or potential of an induced Hawthorne effect may have impacted the behaviour of the probands, and thereby the outcome in both groups. Sweden lacks baseline data on GC uptake, and future research on uptake in an unselected group of HBOC and Lynch families is warranted. The Swedish healthcare context, with tax-funded services and high-cost protection, may limit generalisability to other countries.

A strength of this study is that the HCPs were involved in defining eligible ARRs. In previous studies evaluating cascade GC and testing, the definitions of eligible ARRs vary, and more coherent reporting is warranted. Often, first and second-degree relatives of the proband are considered at risk. Such a simplified definition—without a clinical assessment of the pedigree—could potentially lead to both under- and overestimation of the total number of eligible ARRs. We also think that the objective data source used for the main outcome (ARR’s health record) is a valid proxy for GC uptake as compared to the often-used subjective proband-reported data on ARRs’ uptake. One limitation is our lacking knowledge on the level of understanding and reasoning of those who did not reach out for further counselling. Evidence of the experiences of this under-studied group is limited, and future research on their reasoning is crucial. We acknowledge that the decision of only 12 months of observation time will underestimate the number of eligible ARRs, and we plan a future long-term follow-up study.

## Conclusions

This study, the largest randomised controlled trial evaluating direct contact with ARRs to date, demonstrates that offering direct letters does not significantly increase GC uptake compared to standard family-mediated disclosure. Our findings emphasise the need for a nuanced approach to risk disclosure, recognising that a general healthcare-mediated direct contact approach is not a one-size-fits-all solution and that addressing broader healthcare barriers is crucial for improving overall GC uptake.

## Supplementary information

Study instructions

## Data Availability

Metadata supporting the findings of this study is available at our GitHub repository (https://github.com/RCC6BNH/DIRECT-study-public-repository). Additional data can be made available on reasonable request to the corresponding author. The full dataset cannot be shared due to ethical permit restrictions and concerns regarding identifiability.

## Code availability

The R code used to analyse the data and produce figures is openly available at our GitHub repository (https://github.com/RCC6BNH/DIRECT-study-public-repository).
