# Supplementary material for: Direct letters to relatives at risk of hereditary cancer—a randomised trial on healthcare-assisted versus family-mediated risk disclosure
Source: Eur J Hum Genet. 2025 Jul 31;33(10):1359–67. doi: 10.1038/s41431-025-01922-w (PMC12480553; doi:10.1038/s41431-025-01922-w)
Supplement: Supplementary file 1 — Study instructions [file 41431_2025_1922_MOESM1_ESM.pdf]

Instructions for the DIRECT study

Summary: To be able to randomize participants before the risk disclosure to relatives is discussed, **the invitation** takes place before we know if the patient meets all inclusion criteria (step A). Final **inclusion** in the study will only take place when the results of blood sample analysis are available, the family diagnosis has been established, and the inclusion criteria are met (step B). Treatment depends on which study group the patient is randomized to (Stage C).

Step A – Invitation and study information
Step B – Inclusion and randomisation
Step C – Treatment according to study arm

Step A – Invitation and study information

Anyone who is offered germline analysis for *BRCA1, BRCA2, PALB2, MLH1, MSH2, MSH6, PMS2* can be invited to the DIRECT study. Patients with pathogenic variants in these genes identified through "mainstream testing" are also eligible for the invitation. Research participant information with a consent form is sent home or handed over in two copies (one white and one blue).

If a patient is undergoing testing for variants in the above genes, but you already know for sure that any of the inclusion criteria are not met, the patient should not be asked. If you are unsure whether the patient meets a certain criterion, it is better to ask one too many than one too little.

Due to different inflows for different patients, the invitation procedure may differ (see Table 1).

***Table 1**. Inform about the study and hand over research participant information*

**A. Invitation and study information**			
NB! Only ask individuals over the age of 18, who have the capacity to communicate their own choices.			
**Patients (according to inflow)**	**How do we do it?**	**Who is responsible?**	**Where is it documented?**
Group 1: Recently confirmed **PALB2**, **BRCA1/2 carriers** that have been introduced via clinical testing at another clinic ("mainstream testing")	Research participant information (white and blue) will be sent together with an invitation to visit.	The person who sends the summons.<br>Local manager, with us:<br>____________________	On ***the checklist***<br>In ***the Screening Log*** (or your own customized patient system)
Group 2: Offered "direct testing" of **breast/ovarian/CRC panel** (without family history collection)<br>Group 3: Investigation with genealogy information and offer of **breast/ovarian/CRC panel**<br>Group 4: Carrier testing *(BRCA1, BRCA2, PALB2, MLH1, MSH2, MSH6, PMS2)*	Alt A: Written information sent out with invitation before visits when testing is offered.<br>Alt. B: Info (oral and written) about the study is given in connection with visits when testing is offered.	Alt A: The person who sends the summons.<br>Local manager, with us:<br>____________________<br>Alt B: The one who gives info before testing.<br>Local manager, with us:<br>____________________	On ***the checklist***<br>In ***the Screening Log*** (or your own customized patient system)

Step B – Inclusion and randomisation

Before the post-test counselling, where the significance of the investigation for relatives is discussed, inclusion and exclusion criteria are checked. If the inclusion criteria are met, the person is randomized and study documentation for the patient is started in the CRF forms.

What do I do if the patient wants to participate but the written consent has not been received?
If verbal consent exists, the patient can be randomized. Written consent must then be submitted immediately. Written consent must be received before CRF1 is forwarded (applies to both groups) and/or before direct letters are sent out (intervention group).

Table 2*: Inclusion and randomization of study participants*

**B. Enroll and randomize study patients**			
**Patients**	**How do we do it?**	**Who is responsible?**	**Where to document?**
Any patient who meets all inclusion criteria.	Randomize on a new line in the randomization log.<br>Start the patient's documentation in CRF1 (supplement after visit/test) and prepare CRF2.	Local manager, with us:<br><br>___________________	*On* ***the checklist***<br>*In* ***the Screening Log***<br>*(or your own patient system)*<br>***CRF1 and CRF2***

Step C – Treatment according to each study-arm

The treatment (intervention or current practice) takes place at the post-test counselling, where the significance of the investigation for relatives is discussed. It can happen at;

1) first contact with clinical genetics/cancer genetics clinic (after clinical testing at another clinic `mainstream testing') or
2) The post-test counselling when the results of the blood sample analysis/family investigation is communicated to the patient.

Treatment of all patients enrolled in DIRECT (both study-arms).

1. List at-risk relatives in CRF2 *Note that the list is equally important for both groups.*
Which relatives should be listed?
Familial breast cancer or familial colorectal cancer: Those relatives who, according to the ordinary clinical assessment, are to be offered some form of control program within the next year, i.e. they have reason to contact us within a year to take part in a control program.

Pathogenic variant: Those who, according to ordinary clinical assessment, should be offered testing at this stage in the cascade *(can lead to more individuals being included in the same family, but at a later stage).* If cascade testing is done to find out from which side the variant is inherited, and the clinic follows-up on this, additional at-risk relatives can be added to the CRF2 at a later stage. For example, if cascade testing of the mother is negative, and the father is deceased, then additional relatives on the father's side can be eligible at-risk relatives that have reason to contact a cancer genetic clinic within a year and they should be listed in CRF2.

How should I list the relatives?
With the help of pedigree and information from the patient, you together identify the individuals/at-risk relatives who you have assessed would be eligible for genetic counselling within a year. To find contact information for the relatives, get help from the patient, and search via publicly available websites (e.g. hitta.se/eniro.se/Infotorget.se). If necessary, info can also be filled in after the visit (if, for example, the patient wants to supplement something).
2. Check contact information + survey preference: In the top box of CRF1 there is personal data to reach the patient. Check these and ask if the person wants an electronic questionnaire or a paper questionnaire sent by mail.
3. Remind the patient to answer study questionnaires 1 and 2: Please remind the patient to answer the questionnaires that are sent out immediately (=when CRF1 has arrived in Umeå), and 6 months after the visit.

What do I do if a patient does not want to list relatives or send letters?

Patients can decline to list their families, to send letters (intervention group) or choose to drop out of the study altogether. How we document these situations is clarified in the "*Instructions for drop-outs*" in the study guidelines (available in Swedish).

Table 3*: Treatment of patients in the control group*

<table>
<tr><th colspan="4">Substage C (k) – Post-test counselling for patients <u>in control group</u></th></tr>
<tr><td colspan="4">Treat the patient according to the usual routine.
Fill in CRF2
Check contact details + survey preference and fill in CRF1
Remind them of study questionnaires 1 and 2</td></tr>
<tr><th>Patients (according to inflow)</th><th>When do we give treatment?</th><th>Who is responsible?</th><th>Where is it documented?</th></tr>
<tr><td>Group 1)
Patients via fast-track.</td><td>At the first clinical contact with the clinic – the "first visit".</td><td>The person who gives info during the visit.</td><td rowspan="2">Fill in the date on which the disclosure to the at-risk relatives is discussed with the patient (T=0) in CFR1: F.

Write down preference for the survey in CRF1: A, and check contact information.

Eligible relatives are listed in consultation with the patient in the table on form CRF2: B.</td></tr>
<tr><td>Group 2-4)
Patients undergoing a genetic analysis initiated by the clinic.</td><td>At the post-test counselling*</td><td>The person who gives counselling.</td></tr>
</table>

* If, in connection with the post-test counselling, it is not appropriate to list relatives – the research nurse or other clinically active employee can list relatives by phone afterwards, but still in close proximity in terms of time.

Table 4*: Treatment of patients in the intervention group*

<table>
<tr><th colspan="4">Substage C (i) – Post-test counselling for patient <u>in intervention group</u></th></tr>
<tr><td colspan="4">Treat the patient according to the usual routine.
In addition, offer the opportunity to send out customized DIRECT letters from the clinic to affected relatives (see Manuscript - INTERVENTION).
<u>Ask for consent for direct contact;</u> Ask the patient on the list -for each relative- if the clinic can send a DIRECT letter, one month after the visit. Note Yes or No in the "Proband approved DIRECT letter" box.
Fill in CRF2
Check contact details + survey preference and fill in CRF1
Remind them of study questionnaires 1 and 2</td></tr>
<tr><th>Patients (according to inflow)</th><th>When do we give treatment?</th><th>Who is responsible?</th><th>Where is it documented?</th></tr>
<tr><td>Group 1)
Patients via fast-track.</td><td>At the first clinical contact with the clinic – the "first visit".</td><td>The person who gives info during the visit.</td><td rowspan="2">Fill in the date on which the disclosure to the at-risk relatives is discussed with the patient in CFR1: F.

Write down preference for the survey in CRF1: A, and check contact information.

Eligible relatives are listed in consultation with the patient in the table on form CRF2: B.</td></tr>
<tr><td>Group 2-4)
Patients undergoing a genetic analysis initiated by the clinic.</td><td>At the post-test counselling*</td><td>The person who gives info during the visit.</td></tr>
</table>

<u>After the visit:</u> Submit study documentation to the research nurse

After the appointment visit – submit all study documentation to the local research nurse in charge (***Checklist, CRF1*** and ***CRF2***).

Page 4-8: Detailed instructions for the local research nurse

Locally responsible research nurse ____

Sub-step A. Invitation and study information

Documentation of recruitment

It is important that we can report the reasons behind the fact that invited patients are not included.

Each HCP who recruits for DIRECT at the study site must report each patient they have actively assessed for participation in DIRECT. See separate *Instructions for screening log and randomization* in the study document.

Consent management

If consent has not been received 3 weeks after oral and written information, patients who otherwise continue to meet all inclusion criteria may receive a reminder (see template in the study document). Since so many patients will not fulfil inclusion criteria due to negative results from genetic testing, we no longer recommend a general routine to remind everyone invited of consent – but only in cases where, for example, an eligible patient has expressed interest in the study but there is still no consent submitted prior to a return visit.

Once consent is received; Note it in the checklist, ***screening log*** or your own customized patient system.

Archive consent forms securely and in an orderly manner so that they can be retrieved if necessary.

Consents are archived in ____________________________________

< < WAIT UNTIL YOU HAVE RECEIVED THE PATIENT'S GENETIC ANALYSIS RESULTS > >

Inclusion Criteria (A-E)

Research subjects in the current project meet the inclusion criteria A-E;
A. Investigated at a cancer genetics clinic with the question of hereditary breast, ovarian or colorectal cancer.
B. Are over 18 years of age
C. Has signed consent.
D. Has had any of these four (4) diagnoses established;

1. Familial breast cancer
2. Familial Colorectal Cancer
3. Hereditary breast and ovarian cancer (pathogenic variant in BRC A1/2 gene) or hereditary breast cancer (pathogenic variant in PALB2 gene) or
4. Lynch syndrome (pathogenic variant in MLH1, MSH2, MSH6, PMS2 gene)

E. Has at least one high-risk relative who has not previously received written information about the family's cancer genetic investigation.

Exclusion Criteria (A or B)

a. unable to communicate their own informed decisions (various reasons; language, impairment, etc.)
b. all high-risk relatives currently live outside Sweden.

Explanation of Study-Specific Family Numbers

***Figure 1**. Study-specific numbers of study participants*

Structure of the study-specific numbers in the DIRECT study

The study-specific number for a participating patient is only created when the person is enrolled and randomized to the study. You get the number from the inclusion/randomization tool and is noted on the **CRF1.** It consists of a total of 6 digits in the format X XXX – XX.

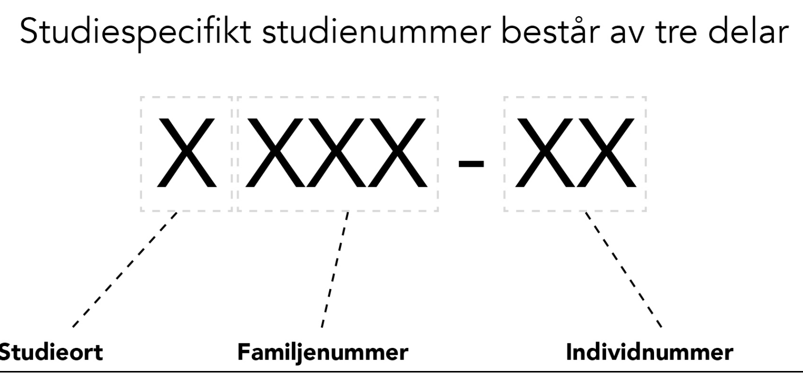

Part B. Inclusion and Randomization

Allocation of treatment group before telephone or appointment

What distinguishes the study groups is the added offer of sending letters to relatives at risk. It is therefore important that allocation to a control or intervention group takes place before post-test counselling. Carry out the randomisation BEFORE post-test counselling. (i.e. for mainstream patients before the first visit to the cancer genetics clinic and in panel testing/carrier testing, before return of results of the analysis).

> When patient belongs to a "new DIRECT family": Fill in the randomization log

Randomization of relatives in a new family

Fill in information about the patient's gender, year of birth and family diagnosis (follow *the Instructions for Screening Log and Randomization* in the study documents.*) In the table, we use a "key" to the diagnosis groups for confidentiality reasons – i.e. the figure for the patient's diagnosis is given:*

1.	Familial breast cancer
2.	Familial colorectal cancer
3.	PALB2 or BRCA1/2
4.	MLH1, MSH2, MSH6, PMS2

When you have filled in the ENTIRE row, your patient is automatically allocated to one of these groups (you can see which one in the blue row on the right, but do not read it until the row is complete, as the algorithm starts "counting" already from you fill in the first box – and this can change when you add information):

1. **control group** (ordinary information about informing at-risk relatives, with the addition that the relatives are listed on form ***CRF2***) or

2. **intervention group** (ordinary information about informing at-risk relatives, with the addition that the relatives are listed on form ***CRF2***, and extended support with an offer of sending DIRECT letters from the healthcare system directly to relatives).

After randomization: Complete the patient's ***CRF1 (box E) and the Checklist.***

> When patient belongs to a family where another relative is already included in DIRECT

Randomization of new patients in the national cascade list

When contacting a new patient (who turns out to have already relatives included in the study), invite the patient to the study if the inclusion criteria are met. To obtain the patient's unique study number, randomize in the "Table for new patients in previously included families" (*separate national excel list*). You will need the study number of the proband and the family's assigned study group.

Fill in the newly recruited patient's ***CRF1, box E, Checklist*** and personal information for ***CRF2***.

Now everything is ready for the patient contact where treatment is administered depending on the study group. Either control (standard care) or intervention (with extra offer of customized DIRECT letters to relatives).

How are documents handled after processing?

1. Complete ***CRF1*** will be sent to the study office as soon as possible after treatment.
2. ***The checklist*** and ***CRF2*** are archived locally in a secure location (for follow-up one year later).

Clarification of which ones to list in CRF2

Table 5: *Clarification of who should be listed in CRF2, **applies to both control and intervention group**, and thus also who should be offered DIRECT letters (if they belong to intervention group).*

Diagnosis	***Listed in CRF2***	***Letter template***	***Age***
No pathogenic variant	*Those who, according to ordinary clinical assessment, would be offered some form of control program AND*<br><br>*- are of age for control programs*	*Familial breast cancer*<br><br>*(See separate tab)*	*Min 18 years*
No pathogenic variant	*Those who, according to ordinary clinical assessment, would be offered some form of control program AND*<br><br>*- are of age for control programs*	*Familial colorectal cancer*<br><br>*(See separate tab)*	*Min 18 years*
Pathogenic variant in BRCA1, BRCA2, PALB2, MLH1, MSH2, MSH6, PMS2	*Those who, according to ordinary clinical assessment, would be offered testing at this stage in the cascade, which means that there are often several individuals who are included in the same family, but at a later stage*<br><br>If cascade testing is done to find out from which side the variant is inherited, and the clinic follows this up through new contact with the index, further supplementation of CRF2 can take place at a later stage*.	*Letter templates depending on the gene and gender of the recipient of the letter. (See separate tab)*	*Min 18 years*

*For example, if cascade testing of the mother is negative, and the father is deceased, then additional relatives on the father's side can be deemed to have reason to contact us within a year and they must then be listed in CRF2 on the individual in question.

INTERVENTION ONLY: Sending DIRECT letters to high-risk relatives (T=1 month)

At time 1 month (counted from the patient's start date in the dark box ***CRF1: F***):

a. After a period of one month from the time t=0 (post-test counselling), DIRECT letters are administered to the eligible relatives at risk.
b. You choose the appropriate DIRECT letter (depending on the patient's family diagnosis, and the gender of the relative).
c. *Note the city, local family number AND study number in the header* ***of each letter*** and sign with the name of the genetic counselor/nurse/doctor who gave the post-test counselling (and/or the person who filled in CRF2 with the patient).
d. *If the patient wanted them to be referred to in the letters: State which relative carried out the investigation. (This is completely voluntary and is decided by the patient).*
e. Letters must be sent to each relative with whom the patient has approved contact, and for whom there is address information. *(The only exception is if the specified high-risk relative has already had contact with the unit from which the letter is to be sent, in these cases note "no" on "DIRECT letter posted to relative" and enter a comment in CRF2 that "the person has already had contact with the unit" and the date of this contact. Also tick the box "Yes" on the question "Have contacted the cancer genetics clinic").*
f. Fill in the right-hand column in each relative's box in ***CRF2: B*** and check that the patient has approved contact via DIRECT letter with that relative.
g. Mail the letter by registered mail to the relatives.
(Depending on the system your hospital has, the routine may vary locally, but general instructions for PacSoft online from PostNord are available from Umeå.)

Time = 12 months after the visit

Calculation of outcome measures and reporting of results (T=12 months)

One year after the patient was notified of the established family diagnosis, the results of communication within the family are examined. The main outcome measure of the study is the ratio between the listed relatives who have contacted a cancer genetics clinic, and the total number of relatives listed for that patient.

Time 12 months – Control group

To sum up the outcome measures, follow these steps:

a. Count the total number of relatives (in CRF2) – fill in the top right row of ***CRF3:C (I).*** If possible, state the gender of the relatives and whether they are a first-degree relative or another relative.
b. Count the total number of relatives for whom there is no contact information (i.e. who are not possible to follow up). Fill it in CRC3:C (II)
c. Check if each relative is in your local patient management system. When meeting, tick "Yes" in ***CRF2: B.***
d. If there is no match, ask based on place of residence, if the relative has been in contact with any of the other cancer genetics clinics in Sweden. If you get a hit here – fill in "Yes". If there is no match also nationally – fill in "No" in ***CRF2: B.***
e. Sum up the total number of people who contacted the healthcare system and complete ***CRF3:C (III).***

Time 12 months – Intervention group

In this group, there will be a little more information to document, otherwise the same routine:

f. Count the total number of relatives (and state the gender and degree of kinship) – fill in ***CRF3:C(I).***
g. Sum from ***CRF2*** the number of approved contacts, ***CRF3:D(I).***
h. If there were relatives who had contacted the clinic BEFORE sending the letter, state how many in ***CRF3:D (II)***
i. Enter the number of DIRECT-letters that were sent out in CRF3:D (III)
j. Then check if the relatives picked up their registered letters – note per individual in ***CRF2***. Sum the number of retrieved messages in ***CRF3:D (IX).***
k. Check if relatives are in your patient management system. Note "Yes" in ***CRF2: B.***
l. If a relative is not available locally, check in the proband journal and/or pedigree if the investigation has been shared with another location (gives an indirect indication that the relative may have contacted them). Note "Yes" in ***CRF2: B.***
m. If a relative is not available locally, and an investigation is not requested, ask based on place of residence if the relative has been in contact with any of the other cancer genetics clinics in Sweden. If yes – fill in "Yes". If there is no match also nationally – fill in "No" in ***CRF2: B.***
n. Sum up the total number of people who contacted the healthcare system and complete ***CRF3:C (III).***

Time: after 12 months

Summary and reporting of the patient's results to Umeå

When you have summarized the results in ***CRF3***, the form must be sent to Umeå. Copy the form and store locally.

For all patients, the two black boxes – ***Outcome measures CRF3:C(I) and CRF3:C(III)*** – must be completed. For study participants in the intervention branch, the entire right column of ***CRF3:D should*** be completed.

***NB! CRF1-CRF3** and **the Checklist** contain sensitive personal data and must be handled in such a way that it complies with current patient data laws and GDPR regulations. We recommend that all CRF forms are handled as other patient data at the clinic. **CRF2** contains raw data for the study, and we therefore ask you to archive this form according to the same regulations that apply to medical records, i.e. at least for ten years.*

Questionnaire send-outs - at 0 and 6 months after inclusion

We follow up all patients in parallel, first with questionnaires (on occasion t=0 months and the second time on t=6 months). *The time starts when the study secretariat in Umeå receives the CRF1 form for the patient.*

At time 0 months: A questionnaire is sent to patients in both groups to be able to compare their experiences of worry and stress over time. The survey is administered either by post or electronically via 1177 My Care Contacts.

At time 6 months: A follow-up questionnaire with the same instrument that was included in T0 is sent to patients in both groups to be able to compare their experiences of worry and stress over time. Like the first survey, it is sent out either by post or electronically.

Version control

Version	Date	News
1.0	200101	First version
2.0	200429	Detailing instructions for the documentation of exclusion and inclusion criteria in the screening log
2.1	200518	Clarifying that also patients with a PV detected by mainstream testing can be included in the study
2.2-2.4	-	No amendments published (2.2, 2.3 and 2,4 working documents)
2.5	201214	Follow-up questionnaire sent out after 6 months instead of after 3 months
2.6	210331	Clarification on documentation of screening log
2.7	220531	Detailing degree of ARRs in CRF3
2.8	220922	Clarification that cascading testing to find out "from which side" the variant comes can lead to CRF2 being supplemented on the individual in question.<br>Comment: During the course of the study, it has become clear that the different study locations have different routines for following up relatives higher up in the cascade. The DIRECT study should not affect current routines, but if the clinic has a routine to follow up this with indexes, additional relatives can be listed in CRF2 at a later stage. Clarifies this also in writing.<br>Regarding inclusion criteria, the parenthesis with lifetime risk behind familial breast cancer and familial colorectal cancer is removed.
2.9	250125	The Swedish version of *Praktiskt genomförande v.2.8 (Uppdaterad 220922) -translated to English*
